# Poly[ethyl­enediammonium [tris­[μ_3_-hydrogenphosphato(2−)]dicadmium] monohydrate]

**DOI:** 10.1107/S1600536810038729

**Published:** 2010-10-02

**Authors:** Abderrazzak Assani, Mohamed Saadi, Lahcen El Ammari

**Affiliations:** a Laboratoire de Chimie du Solide Appliquée, Faculté des Sciences, Université Mohammed V-Agdal, Avenue Ibn Battouta, BP 1014, Rabat, Morocco

## Abstract

The title compound, {(C_2_H_10_N_2_)[Cd_2_(HPO_4_)_3_]·H_2_O}_*n*_, was synthesized under hydro­thermal conditions. The structure of this hybrid compound consists of CdO_6_, CdO_5_ and PO_4_ polyhedra arranged so as to build an anionic inorganic layer, namely [Cd_2_(HPO_4_)_3_]^2−^, parallel to the *ab* plane. The edge-sharing CdO_6_ octa­hedra form infinite chains running along the *a* axis and are linked by CdO_5_ and PO_4_ polyhedra. The ethyl­ene­diammonium cation and the water mol­ecule are located between two adjacent inorganic layers and ensure the cohesion of the structure *via* N—H⋯O and O—H⋯O hydrogen bonds.

## Related literature

For properties of and background to hybride cadmium phosphates, see: Chandrasekhar *et al.* (2010 ▶); Lin *et al.* (2003 ▶, 2005 ▶); Moffat & Jewur (1980 ▶); Qiu *et al.* (2009 ▶). For related structures, see: Cavellec *et al.* (1995 ▶); Assani *et al.* (2010 ▶).
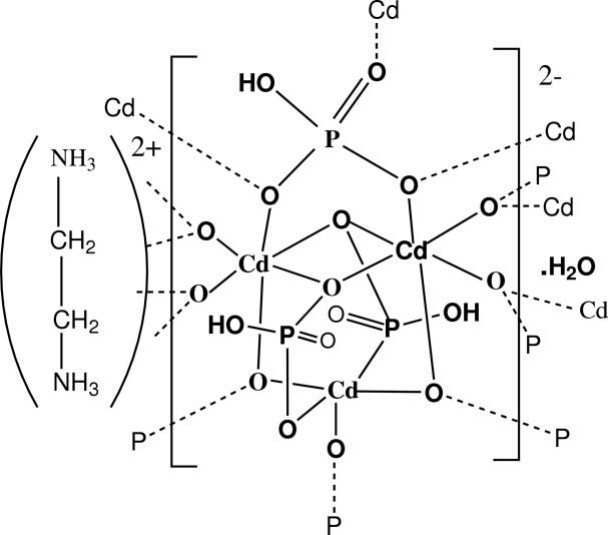


## Experimental

### 

#### Crystal data

(C_2_H_10_N_2_)[Cd_2_(HPO_4_)_3_]·H_2_O
*M* *_r_* = 592.87
Monoclinic, 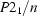

*a* = 6.8203 (1) Å
*b* = 9.5731 (2) Å
*c* = 21.9302 (4) Å
β = 90.274 (1)°
*V* = 1431.84 (4) Å^3^
*Z* = 4
Mo *K*α radiation
μ = 3.38 mm^−1^
*T* = 296 K
0.15 × 0.08 × 0.05 mm

#### Data collection

Bruker X8 APEXII diffractometer
Absorption correction: multi-scan (*SADABS*; Bruker, 2005 ▶) *T*_min_ = 0.730, *T*_max_ = 0.845
21519 measured reflections
4546 independent reflections
4010 reflections with *I* > 2σ(*I*)
*R*_int_ = 0.029

#### Refinement

*R*[*F* ^2^ > 2σ(*F* ^2^)] = 0.022
*wR*(*F* ^2^) = 0.053
*S* = 1.09
4544 reflections
205 parameters
H-atom parameters constrained
Δρ_max_ = 0.81 e Å^−3^
Δρ_min_ = −0.74 e Å^−3^

### 

Data collection: *APEX2* (Bruker, 2005 ▶); cell refinement: *SAINT* (Bruker, 2005 ▶); data reduction: *SAINT*; program(s) used to solve structure: *SHELXS97* (Sheldrick, 2008 ▶); program(s) used to refine structure: *SHELXL97* (Sheldrick, 2008 ▶); molecular graphics: *ORTEP-3 for Windows* (Farrugia,1997 ▶) and *DIAMOND* (Brandenburg, 2006 ▶); software used to prepare material for publication: *WinGX* (Farrugia, 1999 ▶).

## Supplementary Material

Crystal structure: contains datablocks I, global. DOI: 10.1107/S1600536810038729/fk2024sup1.cif

Structure factors: contains datablocks I. DOI: 10.1107/S1600536810038729/fk2024Isup2.hkl

Additional supplementary materials:  crystallographic information; 3D view; checkCIF report

## Figures and Tables

**Table 1:** Hydrogen-bond geometry (Å, °)

*D*—H⋯*A*	*D*—H	H⋯*A*	*D*⋯*A*	*D*—H⋯*A*
N1—H1*A*⋯O9^i^	0.89	1.90	2.774 (2)	165
N1—H1*B*⋯O13^ii^	0.89	2.05	2.895 (3)	159
N1—H1*C*⋯O6^iii^	0.89	1.99	2.866 (2)	170
N2—H2*A*⋯O6^iv^	0.89	1.97	2.823 (2)	160
N2—H2*A*⋯O8^iv^	0.89	2.57	3.243 (3)	133
N2—H2*B*⋯O13^v^	0.89	1.98	2.856 (3)	168
N2—H2*C*⋯O1^iv^	0.89	2.14	2.967 (3)	155
O4—H4⋯O10	0.82	1.97	2.763 (3)	163
O8—H8⋯O10^ii^	0.82	1.81	2.627 (2)	175
O12—H12⋯O5^v^	0.82	1.74	2.547 (2)	166
O13—H13*A*⋯O5	0.86	1.85	2.705 (2)	173
O13—H13*B*⋯O10^vi^	0.86	1.97	2.790 (2)	159
C3—H3*B*⋯O5^iii^	0.97	2.45	3.264 (3)	141
C4—H4*A*⋯O10	0.97	2.59	3.428 (3)	144

Symmetry codes: (i) 
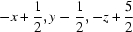
; (ii) 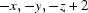; (iii) 
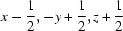
; (iv) 
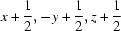
; (v) 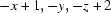; (vi) 
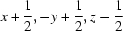
.

**Fig. 1.**
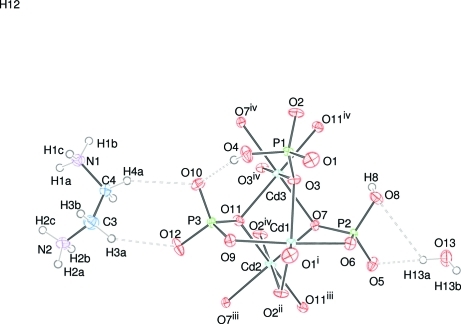
Partial plot of (H3N—CH2—CH2—NH3)Cd2[(HPO4)3].H2O crystal structure. Displacement ellipsoids are drawn at the 50% probability level. Hydrogen bonds are indicated by dashed lines. Symmetry codes: (i) -x + 1/2, y - 1/2, -z + 5/2; (ii) -x, -y, -z + 2; (iii) x - 1/2, -y + 1/2, z + 1/2; (iv) x + 1/2, -y + 1/2, z + 1/2.

**Fig. 2.**
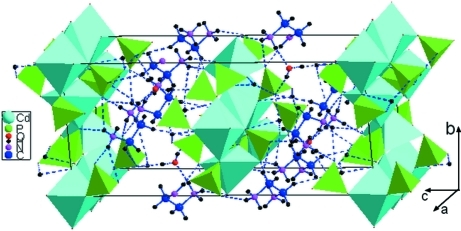
A three-dimensional polyhedral view of the crystal structure of the (H3N—CH2—CH2—NH3)Cd2[(HPO4)3].H2O, showing the stacking of organic and inorganic layers along c axis.

## supplementary crystallographic information

### Comment

Intensive efforts have been greatly devoted to the design of new organic- inorganic materials offering porous and open-framework structures. Such materials are promising for a variety of applications. One class of those materials is the cadmium derived compounds, such as cadmium phosphate by virtue of applications to catalysis (Moffat & Jewur, 1980) and, more recently, cadmium–organic framework used for selective ion sensing (Qiu *et al.* 2009). However, the organically templated cadmium phosphate, a member of such class, remains less investigated. In fact, to our knowledge, the rare compounds isolated in the system Cd – P – organic molecules correspond to Cd(2,2'-bipy)(H_2_PO_4_)_2_ (bipy = bipyridine) (Lin *et al.*, 2003), Cd(phen)(H_2_PO_4_)_2_.H_2_O (phen = 1,10-phenanthroline) (Lin *et al.*, 2005) in addition to those recently published (Chandrasekhar *et al.*, 2010). Consequently, with a view to generate new cadmium hybrid compounds, our interest is focused on the ethylendiamine templated cadmium phosphate with different Cd/P ratio. We present in this work, the hydrothermal synthesis and the structural characterization of the first member of this family with a ratio Cd/P=2/3, namely (H_3_N—CH_2_—CH_2_—NH_3_)Cd_2_[(HPO_4_)_3_].H_2_O compound.

Fig. 1 shows the plot of the asymmetric unit of the title compound with hydrogen bond. A three-dimensional polyhedral view of its crystal structure is represented in Fig. 2. It shows the concatenation of three types of polyhedra: CdO_6_, CdO_5_ and PO_4_. The sharing edge CdO_6_ octahedra form an infinite chain running along the *a* axis. The unshared vertices of the CdO_6_ octahedra are related to PO_4_ tetrahedron and CdO_5_ polyhedron in the way to build a two-dimensional inorganic layer parallel to the plane (a, b). These layers are separated by organic and water molecules as shown in Fig. 2. A similar connectivity is observed in the structure of the two-dimensional iron phosphate templated by ethylenediammonium (C_2_N_2_ H_10_)0.5 [Fe(PO_4_)(OH)] (Cavellec *et al.* 1995).

The cadmium polyhedra show various degres of deformation from idealized geometry. Cd(2)O_6_ and Cd(3)O_6_ octahedra are slightly deformed with Cd–O distances in the range 2.235 (2)–2.333 (2) Å. The Cd(1)O_5_ adopts a distorted trigonal bipyramidal coordination arising from two bidentate ligands (O6—O9; O3—O2^i^) and O1^ii^. The Cd1—O bond lengths vary between 2.166 (2)Å and 2.347 (2) Å. From the three tetrahedrally coordinated phosphorus atoms P1, P2 and P3, the first (P1) shares three O atoms with adjacent cadmium atoms (average distance P—O = 1.519 (2) Å) and possesses one terminal P1—O4 = 1.579 (2) Å. The other phosphorus atoms P2 and P3 are linked to two adjacent cadmium atoms *via* two oxygene atoms (average distance P—O = 1.537 (2) Å) and have two terminal P2═O5 = 1.511 (2) Å and P3═O9 = 1.525 (2) Å and P2—O8 = 1.566 (2) Å and P3—O12 = 1.565 (2) Å bond. The terminal O atoms are involved in hydrogen bonds as show in Table 1. These results corroborate the framework formula and are in close agreement with former study of a similar phosphate (Assani *et al.* 2010).

The ethylenediammonium cation and the water molecules ensure the cohesion of the structure *via* N—H···O and O—H···O hydrogen bonds (Fig. 1, Table 1). Symmetry code: (i) 1 + *x*, *y*, *z* - 1; (ii) -*x*, -*y*, -*z*.

### Experimental

In a typical hydrothermal synthesis, a mixture containing cadmium chloride (CdCl_2_; 0.0917 g), 85 wt % phosphoric acid (H_3_PO_4_; 0.34 ml), ethylenediamine (NH_2_(CH_2_)_2_NH_2_; 0.3 ml), 40 wt % fluoridric acid (HF; 0.1 ml), and water (10 ml), was allowed to react in 23 ml Teflon-lined autoclave under autogeneous pressure at 125°C for tow days. The autoclave were then removed to air and allowed to cool to room temperature. The resulting product was filtered off, washed with deionized water and air dried. The reaction produced colorless parallelepipedic crystals, corresponding to the title compound, (H_3_N—CH_2_—CH_2_—NH_3_)Cd_2_[(HPO_4_)_3_]^.^H_2_O; mixed with some white powder.

### Refinement

All O-bound, N-bound and C-bound H atoms were initially located in a difference map and refined with O—H, N—H and C—H distance restraints of 0.82 (1), (0.86 (1) for the water molecule) Å, 0.89 (1) Å and C–H 0.97 (1) Å, respectively. In a the last cycle they were refined in the riding model approximation with *U*_iso_(H) set to 1.5*U*_eq_(O) or (N) and *U*_iso_(H) set to 1.2 *U*_eq_(C).

The two reflections (0 0 2) and (0 1 1), affected by the beam stop, are eliminated resulting in improved quality of refinement and a significant reduction of *R* and Rw factors. No significant electron density residuals in the difference map.

From the synthetis conditions one might expect an incorporation of F^-^ ions. The distinction by X-ray diffraction between F^-^ and O^2-^ is difficult. However, when the relevant OH positions were replaced by F^-^, a small worsening of the reliability factors was observed. Moreover, the clearly discernible proton positions in the difference Fourier maps point to OH rather than to F. Nevertheless, the existence of a very small amount of F^-^ incorporated in the structure cannot be excluded.

### Crystal data

**Table:**

(C_2_H_10_N_2_)[Cd_2_(HPO_4_)_3_]·H_2_O	*F*(000) = 1144
*M**_r_* = 592.87	*D*_x_ = 2.750 Mg m^−^^3^
Monoclinic, *P*2_1_/*n*	Mo *K*α radiation, λ = 0.71073 Å
Hall symbol: -P 2yn	Cell parameters from 21519 reflections
*a* = 6.8203 (1) Å	θ = 1.9–31.0°
*b* = 9.5731 (2) Å	µ = 3.38 mm^−^^1^
*c* = 21.9302 (4) Å	*T* = 296 K
β = 90.274 (1)°	Prism, colourless
*V* = 1431.84 (4) Å^3^	0.15 × 0.08 × 0.05 mm
*Z* = 4	

### Data collection

**Table:**

Bruker X8 APEXII diffractometer	4546 independent reflections
Radiation source: fine-focus sealed tube	4010 reflections with *I* > 2σ(*I*)
graphite	*R*_int_ = 0.029
φ and ω scans	θ_max_ = 31.0°, θ_min_ = 1.9°
Absorption correction: multi-scan (*SADABS*; Bruker, 2005)	*h* = −9→9
*T*_min_ = 0.730, *T*_max_ = 0.845	*k* = −13→13
21519 measured reflections	*l* = −31→31

### Refinement

**Table:**

Refinement on *F*^2^	Primary atom site location: structure-invariant direct methods
Least-squares matrix: full	Secondary atom site location: difference Fourier map
*R*[*F*^2^ > 2σ(*F*^2^)] = 0.022	Hydrogen site location: inferred from neighbouring sites
*wR*(*F*^2^) = 0.053	H-atom parameters constrained
*S* = 1.09	*w* = 1/[σ^2^(*F*_o_^2^) + (0.0195*P*)^2^ + 1.2601*P*] where *P* = (*F*_o_^2^ + 2*F*_c_^2^)/3
4544 reflections	(Δ/σ)_max_ = 0.002
205 parameters	Δρ_max_ = 0.81 e Å^−^^3^
0 restraints	Δρ_min_ = −0.74 e Å^−^^3^

### Special details

**Table:**

Geometry. All e.s.d.'s (except the e.s.d. in the dihedral angle between two l.s. planes) are estimated using the full covariance matrix. The cell e.s.d.'s are taken into account individually in the estimation of e.s.d.'s in distances, angles and torsion angles; correlations between e.s.d.'s in cell parameters are only used when they are defined by crystal symmetry. An approximate (isotropic) treatment of cell e.s.d.'s is used for estimating e.s.d.'s involving l.s. planes.
Refinement. Refinement of *F*^2^ against ALL reflections. The weighted *R*-factor *wR* and goodness of fit *S* are based on *F*^2^, conventional *R*-factors *R* are based on *F*, with *F* set to zero for negative *F*^2^. The threshold expression of *F*^2^ > σ(*F*^2^) is used only for calculating *R*-factors(gt) *etc*. and is not relevant to the choice of reflections for refinement. *R*-factors based on *F*^2^ are statistically about twice as large as those based on *F*, and *R*- factors based on ALL data will be even larger.

### Fractional atomic coordinates and isotropic or equivalent isotropic displacement parameters (Å^2^)

**Table:**

	*x*	*y*	*z*	*U*_iso_*/*U*_eq_	
Cd1	0.265141 (19)	0.322444 (16)	0.995676 (6)	0.01225 (4)	
Cd2	0.5000	0.0000	1.0000	0.01160 (5)	
Cd3	0.0000	0.0000	1.0000	0.01155 (5)	
P1	−0.23831 (7)	0.31181 (5)	1.01284 (2)	0.01023 (9)	
P2	0.26500 (7)	0.14162 (5)	0.87635 (2)	0.00985 (9)	
P3	0.22616 (7)	0.16014 (5)	1.11897 (2)	0.01106 (10)	
O1	−0.2503 (2)	0.45600 (17)	0.98506 (8)	0.0231 (3)	
O2	−0.4243 (2)	0.23029 (16)	0.99783 (7)	0.0165 (3)	
O3	−0.0553 (2)	0.23344 (15)	0.99202 (7)	0.0135 (3)	
O4	−0.2219 (2)	0.3313 (2)	1.08413 (7)	0.0252 (4)	
H4	−0.1629	0.2647	1.0989	0.038*	
O5	0.4393 (2)	0.10843 (17)	0.83666 (7)	0.0189 (3)	
O6	0.2610 (2)	0.29729 (16)	0.89393 (7)	0.0160 (3)	
O7	0.2583 (2)	0.05068 (17)	0.93438 (6)	0.0148 (3)	
O8	0.0698 (2)	0.11514 (17)	0.84012 (8)	0.0209 (3)	
H8	0.0409	0.0321	0.8421	0.031*	
O9	0.2659 (2)	0.30932 (16)	1.09801 (7)	0.0163 (3)	
O10	0.0213 (2)	0.14966 (16)	1.14671 (7)	0.0181 (3)	
O11	0.2469 (2)	0.05774 (16)	1.06531 (6)	0.0150 (3)	
O12	0.3816 (3)	0.12463 (19)	1.16923 (7)	0.0243 (4)	
H12	0.4319	0.0488	1.1617	0.036*	
O13	0.3841 (3)	0.12448 (19)	0.71465 (7)	0.0280 (4)	
H13A	0.3975	0.1271	0.7536	0.042*	
H13B	0.4199	0.2058	0.7023	0.042*	
N1	−0.0232 (3)	−0.0197 (2)	1.33813 (9)	0.0195 (4)	
H1A	0.0428	−0.0751	1.3635	0.029*	
H1B	−0.1147	−0.0691	1.3187	0.029*	
H1C	−0.0800	0.0486	1.3592	0.029*	
N2	0.4080 (3)	0.0696 (2)	1.35973 (9)	0.0232 (4)	
H2A	0.5009	0.1303	1.3703	0.035*	
H2B	0.4613	−0.0005	1.3389	0.035*	
H2C	0.3509	0.0362	1.3931	0.035*	
C4	0.1140 (3)	0.0414 (3)	1.29303 (10)	0.0248 (5)	
H4A	0.0389	0.0907	1.2621	0.030*	
H4B	0.1846	−0.0335	1.2731	0.030*	
C3	0.2594 (4)	0.1406 (3)	1.32117 (12)	0.0268 (5)	
H3A	0.3257	0.1912	1.2889	0.032*	
H3B	0.1895	0.2082	1.3458	0.032*	

### Atomic displacement parameters (Å^2^)

**Table:**

	*U*^11^	*U*^22^	*U*^33^	*U*^12^	*U*^13^	*U*^23^
Cd1	0.01344 (7)	0.00955 (7)	0.01375 (7)	0.00046 (5)	−0.00049 (5)	−0.00069 (5)
Cd2	0.01005 (8)	0.00937 (10)	0.01536 (9)	0.00148 (6)	−0.00147 (7)	−0.00025 (7)
Cd3	0.01014 (8)	0.00897 (10)	0.01556 (9)	−0.00122 (6)	−0.00001 (7)	−0.00143 (7)
P1	0.0102 (2)	0.0071 (2)	0.0134 (2)	0.00079 (16)	−0.00040 (17)	−0.00148 (18)
P2	0.0124 (2)	0.0084 (2)	0.0088 (2)	0.00099 (16)	0.00007 (17)	0.00108 (17)
P3	0.0135 (2)	0.0097 (2)	0.0100 (2)	0.00022 (17)	−0.00054 (17)	−0.00105 (18)
O1	0.0288 (8)	0.0076 (7)	0.0331 (9)	0.0025 (6)	0.0040 (7)	0.0018 (7)
O2	0.0100 (6)	0.0113 (7)	0.0283 (8)	−0.0011 (5)	−0.0020 (6)	−0.0018 (6)
O3	0.0110 (6)	0.0097 (7)	0.0198 (7)	0.0006 (5)	0.0021 (5)	−0.0019 (6)
O4	0.0267 (8)	0.0332 (10)	0.0155 (7)	0.0130 (7)	−0.0031 (6)	−0.0069 (7)
O5	0.0225 (7)	0.0184 (8)	0.0160 (7)	0.0057 (6)	0.0046 (6)	0.0026 (6)
O6	0.0202 (7)	0.0117 (7)	0.0161 (7)	0.0005 (5)	0.0008 (6)	−0.0004 (6)
O7	0.0123 (6)	0.0202 (8)	0.0119 (6)	0.0006 (5)	−0.0012 (5)	0.0058 (6)
O8	0.0220 (7)	0.0166 (8)	0.0240 (8)	−0.0046 (6)	−0.0097 (6)	0.0046 (7)
O9	0.0208 (7)	0.0116 (7)	0.0166 (7)	−0.0020 (6)	−0.0014 (6)	0.0011 (6)
O10	0.0176 (7)	0.0168 (8)	0.0200 (7)	−0.0017 (6)	0.0059 (6)	−0.0025 (6)
O11	0.0131 (6)	0.0188 (8)	0.0131 (6)	0.0007 (5)	−0.0004 (5)	−0.0068 (6)
O12	0.0313 (8)	0.0234 (9)	0.0181 (7)	0.0118 (7)	−0.0120 (7)	−0.0050 (7)
O13	0.0393 (10)	0.0281 (10)	0.0167 (8)	−0.0095 (8)	−0.0031 (7)	0.0015 (7)
N1	0.0172 (8)	0.0191 (10)	0.0222 (9)	0.0000 (7)	−0.0002 (7)	0.0029 (8)
N2	0.0211 (9)	0.0230 (11)	0.0254 (10)	−0.0078 (7)	−0.0016 (8)	0.0009 (8)
C4	0.0244 (10)	0.0327 (14)	0.0172 (10)	−0.0061 (10)	−0.0016 (8)	0.0055 (10)
C3	0.0261 (11)	0.0228 (12)	0.0314 (12)	−0.0052 (9)	−0.0038 (10)	0.0072 (10)

### Geometric parameters (Å, °)

**Table:**

Cd1—O1^i^	2.1651 (17)	P2—O8	1.5676 (16)
Cd1—O6	2.2443 (15)	P3—O9	1.5250 (16)
Cd1—O9	2.2477 (15)	P3—O10	1.5301 (15)
Cd1—O2^ii^	2.2950 (14)	P3—O11	1.5386 (15)
Cd1—O3	2.3464 (14)	P3—O12	1.5631 (16)
Cd1—Cd2	3.47873 (16)	O1—Cd1^i^	2.1652 (17)
Cd2—O7^iii^	2.2362 (14)	O2—Cd2^v^	2.2648 (15)
Cd2—O7	2.2362 (14)	O2—Cd1^v^	2.2950 (14)
Cd2—O2^iv^	2.2648 (15)	O4—H4	0.8200
Cd2—O2^ii^	2.2648 (15)	O8—H8	0.8200
Cd2—O11^iii^	2.3150 (13)	O12—H12	0.8200
Cd2—O11	2.3150 (13)	O13—H13A	0.8599
Cd2—Cd3	3.41020 (15)	O13—H13B	0.8598
Cd2—Cd1^iii^	3.47868 (16)	N1—C4	1.485 (3)
Cd3—O3^iv^	2.2729 (15)	N1—H1A	0.8900
Cd3—O3	2.2729 (15)	N1—H1B	0.8900
Cd3—O11^iv^	2.2738 (14)	N1—H1C	0.8900
Cd3—O11	2.2739 (14)	N2—C3	1.482 (3)
Cd3—O7	2.3312 (13)	N2—H2A	0.8900
Cd3—O7^iv^	2.3312 (13)	N2—H2B	0.8900
P1—O1	1.5109 (18)	N2—H2C	0.8900
P1—O2	1.5238 (15)	C4—C3	1.503 (3)
P1—O3	1.5282 (14)	C4—H4A	0.9700
P1—O4	1.5779 (16)	C4—H4B	0.9700
P2—O5	1.5104 (15)	C3—H3A	0.9700
P2—O6	1.5395 (16)	C3—H3B	0.9700
P2—O7	1.5427 (15)		
			
O1^i^—Cd1—O6	107.38 (6)	O3—Cd3—Cd2	99.52 (3)
O1^i^—Cd1—O9	81.94 (6)	O11^iv^—Cd3—Cd2	137.54 (3)
O6—Cd1—O9	170.62 (6)	O11—Cd3—Cd2	42.47 (3)
O1^i^—Cd1—O2^ii^	114.61 (6)	O7—Cd3—Cd2	40.65 (3)
O6—Cd1—O2^ii^	89.22 (6)	O7^iv^—Cd3—Cd2	139.35 (3)
O9—Cd1—O2^ii^	87.72 (6)	O3^iv^—Cd3—Cd2^v^	99.52 (3)
O1^i^—Cd1—O3	108.55 (6)	O3—Cd3—Cd2^v^	80.48 (3)
O6—Cd1—O3	85.39 (5)	O11^iv^—Cd3—Cd2^v^	42.46 (3)
O9—Cd1—O3	90.67 (5)	O11—Cd3—Cd2^v^	137.53 (3)
O2^ii^—Cd1—O3	136.09 (5)	O7—Cd3—Cd2^v^	139.35 (3)
O1^i^—Cd1—Cd2	152.36 (5)	O7^iv^—Cd3—Cd2^v^	40.65 (3)
O6—Cd1—Cd2	86.30 (4)	Cd2—Cd3—Cd2^v^	180.0
O9—Cd1—Cd2	85.66 (4)	O1—P1—O2	109.71 (9)
O2^ii^—Cd1—Cd2	39.96 (4)	O1—P1—O3	111.77 (9)
O3—Cd1—Cd2	96.16 (4)	O2—P1—O3	111.37 (9)
O7^iii^—Cd2—O7	180.0	O1—P1—O4	107.17 (10)
O7^iii^—Cd2—O2^iv^	86.72 (6)	O2—P1—O4	109.25 (10)
O7—Cd2—O2^iv^	93.28 (6)	O3—P1—O4	107.44 (9)
O7^iii^—Cd2—O2^ii^	93.28 (6)	O5—P2—O6	111.28 (9)
O7—Cd2—O2^ii^	86.72 (6)	O5—P2—O7	112.53 (9)
O2^iv^—Cd2—O2^ii^	180.00 (7)	O6—P2—O7	109.82 (9)
O7^iii^—Cd2—O11^iii^	78.28 (5)	O5—P2—O8	110.02 (10)
O7—Cd2—O11^iii^	101.72 (5)	O6—P2—O8	105.51 (9)
O2^iv^—Cd2—O11^iii^	87.22 (5)	O7—P2—O8	107.37 (8)
O2^ii^—Cd2—O11^iii^	92.79 (5)	O9—P3—O10	110.20 (9)
O7^iii^—Cd2—O11	101.72 (5)	O9—P3—O11	110.43 (9)
O7—Cd2—O11	78.28 (5)	O10—P3—O11	110.49 (9)
O2^iv^—Cd2—O11	92.78 (5)	O9—P3—O12	107.17 (10)
O2^ii^—Cd2—O11	87.21 (5)	O10—P3—O12	108.84 (9)
O11^iii^—Cd2—O11	180.00 (7)	O11—P3—O12	109.64 (9)
O7^iii^—Cd2—Cd3^ii^	42.78 (3)	P1—O1—Cd1^i^	144.97 (11)
O7—Cd2—Cd3^ii^	137.23 (3)	P1—O2—Cd2^v^	133.13 (9)
O2^iv^—Cd2—Cd3^ii^	103.19 (4)	P1—O2—Cd1^v^	125.08 (9)
O2^ii^—Cd2—Cd3^ii^	76.81 (4)	Cd2^v^—O2—Cd1^v^	99.44 (5)
O11^iii^—Cd2—Cd3^ii^	41.54 (4)	P1—O3—Cd3	126.52 (8)
O11—Cd2—Cd3^ii^	138.46 (4)	P1—O3—Cd1	125.00 (8)
O7^iii^—Cd2—Cd3	137.22 (3)	Cd3—O3—Cd1	101.56 (5)
O7—Cd2—Cd3	42.77 (3)	P1—O4—H4	109.5
O2^iv^—Cd2—Cd3	76.81 (4)	P2—O6—Cd1	110.65 (8)
O2^ii^—Cd2—Cd3	103.19 (4)	P2—O7—Cd2	128.95 (8)
O11^iii^—Cd2—Cd3	138.46 (4)	P2—O7—Cd3	130.69 (8)
O11—Cd2—Cd3	41.54 (4)	Cd2—O7—Cd3	96.58 (5)
Cd3^ii^—Cd2—Cd3	180.0	P2—O8—H8	109.5
O7^iii^—Cd2—Cd1^iii^	56.77 (4)	P3—O9—Cd1	110.71 (8)
O7—Cd2—Cd1^iii^	123.23 (4)	P3—O11—Cd3	124.47 (8)
O2^iv^—Cd2—Cd1^iii^	40.60 (4)	P3—O11—Cd2	134.18 (8)
O2^ii^—Cd2—Cd1^iii^	139.40 (4)	Cd3—O11—Cd2	95.99 (5)
O11^iii^—Cd2—Cd1^iii^	57.35 (4)	P3—O12—H12	109.5
O11—Cd2—Cd1^iii^	122.65 (4)	H13A—O13—H13B	105.0
Cd3—Cd2—Cd1^iii^	117.408 (2)	C4—N1—H1A	109.5
O7^iii^—Cd2—Cd1	123.23 (4)	C4—N1—H1B	109.5
O7—Cd2—Cd1	56.77 (4)	H1A—N1—H1B	109.5
O2^iv^—Cd2—Cd1	139.40 (4)	C4—N1—H1C	109.5
O2^ii^—Cd2—Cd1	40.60 (4)	H1A—N1—H1C	109.5
O11^iii^—Cd2—Cd1	122.65 (4)	H1B—N1—H1C	109.5
O11—Cd2—Cd1	57.35 (4)	C3—N2—H2A	109.5
Cd3—Cd2—Cd1	62.592 (2)	C3—N2—H2B	109.5
Cd1^iii^—Cd2—Cd1	180.0	H2A—N2—H2B	109.5
O3^iv^—Cd3—O3	179.999 (1)	C3—N2—H2C	109.5
O3^iv^—Cd3—O11^iv^	86.08 (5)	H2A—N2—H2C	109.5
O3—Cd3—O11^iv^	93.93 (5)	H2B—N2—H2C	109.5
O3^iv^—Cd3—O11	93.92 (5)	N1—C4—C3	113.1 (2)
O3—Cd3—O11	86.08 (5)	N1—C4—H4A	109.0
O11^iv^—Cd3—O11	180.00 (8)	C3—C4—H4A	109.0
O3^iv^—Cd3—O7	97.28 (5)	N1—C4—H4B	109.0
O3—Cd3—O7	82.72 (5)	C3—C4—H4B	109.0
O11^iv^—Cd3—O7	102.80 (5)	H4A—C4—H4B	107.8
O11—Cd3—O7	77.21 (5)	N2—C3—C4	113.1 (2)
O3^iv^—Cd3—O7^iv^	82.73 (5)	N2—C3—H3A	109.0
O3—Cd3—O7^iv^	97.27 (5)	C4—C3—H3A	109.0
O11^iv^—Cd3—O7^iv^	77.20 (5)	N2—C3—H3B	109.0
O11—Cd3—O7^iv^	102.79 (5)	C4—C3—H3B	109.0
O7—Cd3—O7^iv^	180.00 (4)	H3A—C3—H3B	107.8
O3^iv^—Cd3—Cd2	80.48 (3)		

Symmetry codes: (i) −*x*, −*y*+1, −*z*+2; (ii) *x*+1, *y*, *z*; (iii) −*x*+1, −*y*, −*z*+2; (iv) −*x*, −*y*, −*z*+2; (v) *x*−1, *y*, *z*.

### Hydrogen-bond geometry (Å, °)

**Table:**

*D*—H···*A*	*D*—H	H···*A*	*D*···*A*	*D*—H···*A*
N1—H1A···O9^vi^	0.89	1.90	2.774 (2)	165
N1—H1B···O13^iv^	0.89	2.05	2.895 (3)	159
N1—H1C···O6^vii^	0.89	1.99	2.866 (2)	170
N2—H2A···O6^viii^	0.89	1.97	2.823 (2)	160
N2—H2A···O8^viii^	0.89	2.57	3.243 (3)	133
N2—H2B···O13^iii^	0.89	1.98	2.856 (3)	168
N2—H2C···O1^viii^	0.89	2.14	2.967 (3)	155
O4—H4···O10	0.82	1.97	2.763 (3)	163
O8—H8···O10^iv^	0.82	1.81	2.627 (2)	175
O12—H12···O5^iii^	0.82	1.74	2.547 (2)	166
O13—H13A···O5	0.86	1.85	2.705 (2)	173
O13—H13B···O10^ix^	0.86	1.97	2.790 (2)	159
C3—H3B···O5^vii^	0.97	2.45	3.264 (3)	141
C4—H4A···O10	0.97	2.59	3.428 (3)	144

Symmetry codes: (vi) −*x*+1/2, *y*−1/2, −*z*+5/2; (iv) −*x*, −*y*, −*z*+2; (vii) *x*−1/2, −*y*+1/2, *z*+1/2; (viii) *x*+1/2, −*y*+1/2, *z*+1/2; (iii) −*x*+1, −*y*, −*z*+2; (ix) *x*+1/2, −*y*+1/2, *z*−1/2.
